# Supplementary material for: Tetrahydrocurcumin Has Similar Anti-Amyloid Properties as Curcumin: In Vitro Comparative Structure-Activity Studies
Source: Antioxidants (Basel). 2021 Oct 11;10(10):1592. doi: 10.3390/antiox10101592 (PMC8533373; doi:10.3390/antiox10101592)
Supplement: Supplementary file 1 [file antioxidants-10-01592-s001.zip › antioxidants-1350824-supplementary.pdf]

antioxidants MDPI

Supplemental materials

Table S1. Chemicals and their sources.

Chemicals	Source	Catalog number	Address
Curcumin	Sigma	C1386-50G	St. Louis, MO
Bisdemethoxycurcumin	Sigma	B6938-25MG	St. Louis, MO
Demethoxycurucmin	Sigma	D7696-25MG	St. Louis, MO
Tetrahydrocurcumin	Sigma	SMB00370-1G	St. Louis, MO
Hexafluoroisopropanol	Sigma	8045150025	St. Louis, MO
Aβ42	Sigma	A9810-.1MG	St. Louis, MO
6E10	BioLegend	803001	San Diego, CA
A11	Thermo Fisher Scientific	AHB0052	Waltham, MA
OC	Millipore-Sigma	AB2286	Burlington, MA
Caspase-3	Cell Signaling Technology	14220	Danvers, MA
Chemiluminescent kit	ThermoScientific	34096	Waltham, MA
Silver stain kit	Thermo Fisher Scientific	24612	Waltham, MA
N2A cells	ATCC	ATCC® CCL-131	Manassas, VA
SH-SY5Y cells	ATCC	ATCC®CRL-2266	Manassas, VA
Eagle's Minimum Essential Media	GIBCO	ATCC	Manassas, VA
DMEM: F12K	GIBCO	11320-033	Manassas, VA
Fetal bovine serum	GIBCO	A4766801	Manassas, VA
MTT	Sigma	M2128-5G	St. Louis, MO
96-well plates	Denville Scientific Inc	2019001	Metuchen, NJ
Ammonium persulphate	Fisher Scientific	BP179-25	Waltham, MA
RuBpy	Sigma	754730	St. Louis, MO
PVDF membrane	Millipore	IPVH00010	Bedford, MA
β-mercaptoethanol	GIBCO	21985	Manassas, VA
Copper grid	Sigma	G4901-1VL	St. Louis, MO
Glutaraldehyde	Sigma	G5882	St. Louis, MO
BCA kit	Thermo Fisher Scientific	BCA1-1KT	Waltham, MA

Table S2. Different chemicals and their amounts used in PICUP reaction. APS: ammonium per sulphate; RuBpy: ruthenium bipyridine.

Aβ42 (μL)	APS (μL)	RuBpy (μL)	Light exposure (μL)	β-mercaptoethanol
18	1	1	1	10
38	2	2	1	20

Table S3. Binding affinity levels in Cur derivatives when interacting with Aβ40 and Aβ42. The binding affinity were calculated using Auto Dock Vina software. Note that the keto and enol form of curcumin were more likely interacted both Aβ40 and 42. Also, the more bioavailable THC has stronger binding affinity for the Aβ42 over Aβ40.

Derivatives	Interactions with amino acids of Aβ40	Binding affinity(kcal/mol)	Interactions with amino acids of Aβ42	Binding affinity (kcal/mol)
KCur	Asp-1, Gly-9, Tyr-10, Lys-16, Leu-17, Phe-20	-6.3	Glu-3, His-6, Tyr-10, His-13	-5.5
ECur	Glu-3, His-6, Tyr-10, His-13, His-14	-6.1	Glu-3, His-6, Tyr-10, His-14	-5.4
BDMC	Asp-1, Glu-3, Val-12, His-13, Lys-16, Phe-20	-5.9	His-6, Tyr-10	-5.2
DMC	Glu-3, Gly-9, Val-12, His-13, Lys-16	-5.5	Ser-8, Val-12, Lys-16, Phe-19	-5
THC	Asp-1, Glu-3, Lys-16, Phe-20	-4.8	Glu-3, His-6, Asp-7, Tyr-10, His-13, His-14	-5

Table S4. Hydrogen bond formation of different amino acids of Aβ40 and Aβ42 when interacted with KCur and THC.

**Aβ-40-Kcur**			**Aβ-40-THC Simulation**		
Found	20	H-bonds	Found	28	H-bonds.
Donor	Acceptor	Occupancy	Donor	Acceptor	Occupancy
LIG1-Side	ASP7-Main	0.01%	ASP1-Main	LIG1-Side	0.36%
LIG1-Side	ASP1-Main	0.29%	LIG1-Side	ASP7-Main	0.02%
LIG1-Side	GLU3-Side	0.01%	HSD6-Main	LIG1-Side	0.20%
HSD13-Side	LIG1-Side	0.07%	LIG1-Side	PHE4-Main	0.01%
LIG1-Side	ALA2-Main	0.07%	LYS16-Side	LIG1-Side	0.29%
ASN27-Side	LIG1-Side	0.26%	SER8-Side	LIG1-Side	0.05%
LYS16-Side	LIG1-Side	0.22%	ALA2-Main	LIG1-Side	0.05%
LIG1-Side	VAL24-Main	0.10%	GLY9-Main	LIG1-Side	0.13%
LYS28-Side	LIG1-Side	0.49%	TYR10-Side	LIG1-Side	0.10%
LIG1-Side	ASN27-Side	0.02%	LIG1-Side	GLY9-Main	0.04%
TYR10-Side	LIG1-Side	0.49%	SER8-Main	LIG1-Side	0.11%
PHE4-Main	LIG1-Side	0.65%	LIG1-Side	ASP7-Side	0.20%

LIG1-Side	HSD6-Side	0.04%	LIG1-Side	ASP23-Side	0.36%
LIG1-Side	ALA21-Main	0.06%	LIG1-Side	TYR10-Side	0.02%
LIG1-Side	GLU22-Side	0.12%	ASN27-Side	LIG1-Side	0.04%
LIG1-Side	TYR10-Main	0.01%	LIG1-Side	ASN27-Side	0.01%
SER26-Side	LIG1-Side	0.04%	LIG1-Side	SER8-Side	0.12%
ASP1-Main	LIG1-Side	0.01%	LIG1-Side	GLU11-Side	0.25%
TYR10-Main	LIG1-Side	0.04%	LIG1-Side	HSD14-Side	0.05%
GLY9-Main	LIG1-Side	0.01%	LIG1-Side	GLU3-Main	0.18%
			ARG5-Side	LIG1-Side	0.06%
			ARG5-Main	LIG1-Side	0.10%
			TYR10-Main	LIG1-Side	0.22%
			GLU11-Main	LIG1-Side	0.10%
			ASP7-Main	LIG1-Side	0.17%
			LIG1-Side	LYS16-Main	0.36%
			LIG1-Side	GLN15-Main	0.04%
			LYS16-Main	LIG1-Side	0.67%
**Aβ-42-KCur Simulation**			**Aβ-42-THC Simulation**		
Found	19	H-bonds	Found	25	H-bonds.
Donor	Acceptor	Occupancy	Donor	Acceptor	Occupancy
LIG1-Side	GLU3-Side	0.06%	HSD6-Side	LIG1-Side	0.11%
ASP1-Main	LIG1-Side	0.05%	LIG1-Side	HSD14-Side	0.05%
LIG1-Side	GLN15-Side	0.26%	LIG1-Side	GLU3-Side	0.05%
LIG1-Side	GLU11-Side	0.01%	LIG1-Side	TYR10-Main	0.01%
GLN15-Side	LIG1-Side	0.02%	HSD14-Side	LIG1-Side	0.44%
HSD6-Side	LIG1-Side	0.06%	LIG1-Side	LEU17-Main	0.01%
LIG1-Side	ALA21-Main	0.01%	LIG1-Side	ASP1-Side	0.13%
LIG1-Side	HSD6-Main	0.01%	TYR10-Side	LIG1-Side	0.01%

LIG1-Side	ILE41-Main	0.10%	LIG1-Side	HSD13-Side	0.10%
LIG1-Side	ASP7-Side	0.02%	LIG1-Side	SER8-Side	0.01%
LIG1-Side	VAL40-Main	0.09%	LIG1-Side	VAL36-Main	0.01%
LIG1-Side	VAL39-Main	0.17%	LIG1-Side	ASP1-Main	0.04%
HSD6-Main	LIG1-Side	0.01%	ALA30-Main	LIG1-Side	0.04%
LIG1-Side	LYS28-Main	0.02%	ASN27-Side	LIG1-Side	0.07%
ARG5-Side	LIG1-Side	0.11%	LIG1-Side	ASN27-Side	0.12%
TYR10-Side	LIG1-Side	0.03%	LYS28-Main	LIG1-Side	0.05%
LIG1-Side	ALA42-Side	0.78%	LIG1-Side	SER26-Main	0.02%
ALA42-Main	LIG1-Side	0.14%	LYS16-Side	LIG1-Side	0.30%
TYR10-Main	LIG1-Side	0.02%	LIG1-Side	GLU22-Side	0.32%
			LIG1-Side	ALA42-Side	0.01%
			ILE31-Main	LIG1-Side	0.04%
			LIG1-Side	HSD6-Side	0.01%
			ARG5-Side	LIG1-Side	0.28%
			LIG1-Side	HSD6-Main	0.06%
			LIG1-Side	VAL40-Main	0.01%

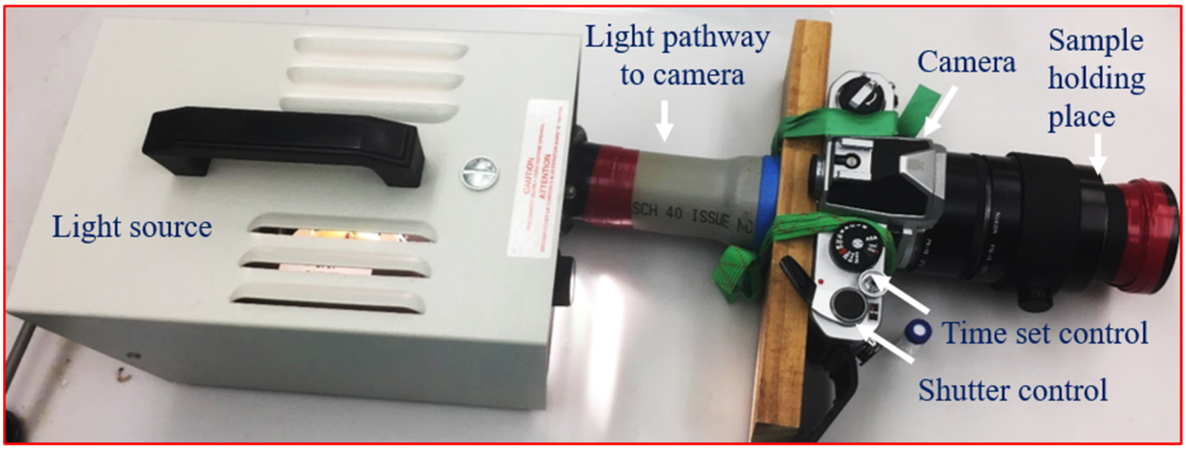

Figure S1. Camera apparatus used to irradiate the protein mixture to perform photo-induced cross-linking of unmodified protein (PICUP). It includes a light source and a camera with shutter and time control.

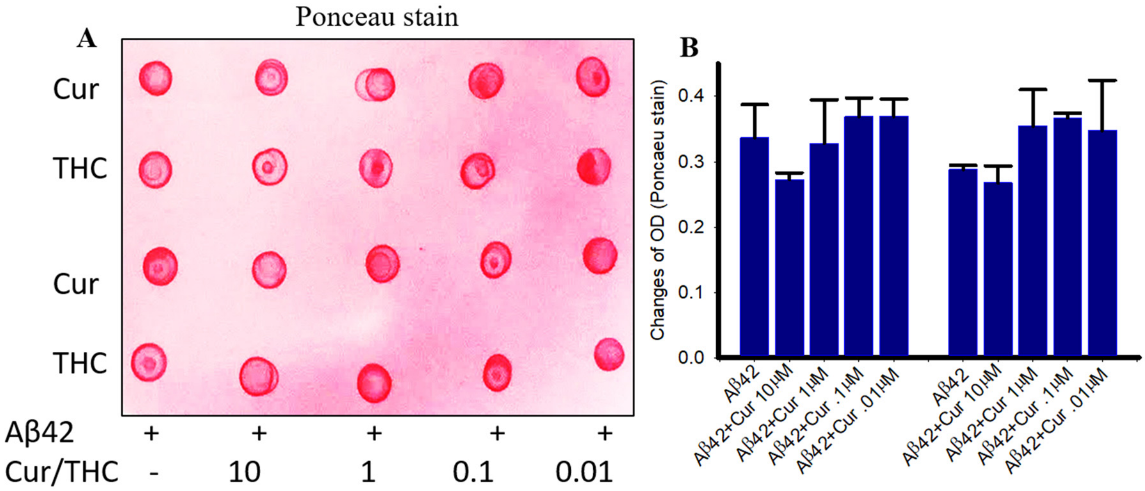

Figure S2. Ponceau stained dot blot. After loading 10 μL of Aβ42 peptide solution from each group on the nitrocellulose membrane, the blot was stained with Ponceau stain. **A:** Representative image of dot blot after Ponceau stain. **B:** No significant changes in optical density among the groups were observed.

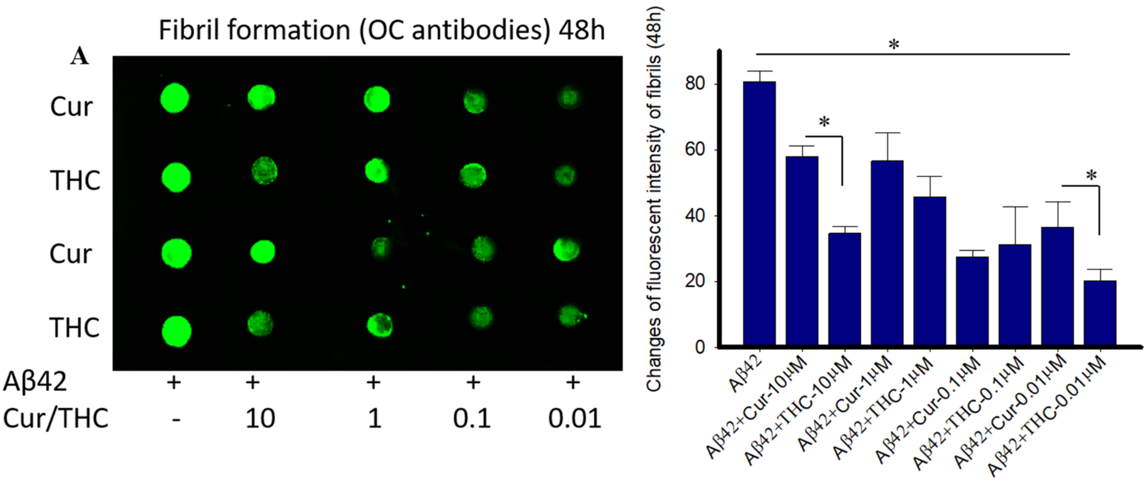

Figure S3. THC inhibited Aβ42 fibrils formation greater than Cur. Aβ42 peptide (40 μM) was disaggregated with HFIP and dissolved in 60 mM of NaOH and diluted with PBS (pH7.4) and incubated for 24-48 h in presence or absence of different Cur-derivatives (1μM). Ten microliters of peptide from each group were spotted on nitrocellulose membrane, incubated with OC antibodies and probed with anti-rabbit antibodies conjugated with fluorophore 488 and the fluorescent intensity was recorded using a gel documentation system. **A**: Representative dot blot images image after Aβ42 was treated for 48 h with different concentrations of Cur and THC (in μM: 10, 1, 0.1 and 0.001). **B:** THC showed greater inhibition for Aβ42 fibril formation than Cur. *p<0.05 in comparison to untreated group and #p<0.05 in comparison to Cut-treated groups.
